# Paleogenomics illuminates the evolutionary history of the extinct Holocene “horned” crocodile of Madagascar, *Voay robustus*

**DOI:** 10.1038/s42003-021-02017-0

**Published:** 2021-04-27

**Authors:** E. Hekkala, J. Gatesy, A. Narechania, R. Meredith, M. Russello, M. L. Aardema, E. Jensen, S. Montanari, C. Brochu, M. Norell, G. Amato

**Affiliations:** 1 Department of Biological Sciences, Fordham University, Bronx, NY USA; 2 American Museum of Natural History, New York, NY USA; 3 Montclair State University, Montclair, NJ USA; 4 University of British Columbia, Department of Biology, Kelowna, BC Canada; 5 Newcastle University, School of Natural and Environmental Sciences Ecology Group, Newcastle, UK; 6 University of Iowa, Department of Geosciences, Iowa City, IA USA

**Keywords:** Palaeontology, Zoology

## Abstract

Ancient DNA is transforming our ability to reconstruct historical patterns and mechanisms shaping modern diversity and distributions. In particular, molecular data from extinct Holocene island faunas have revealed surprising biogeographic scenarios. Here, we recovered partial mitochondrial (mt) genomes for 1300–1400 year old specimens (*n* = 2) of the extinct “horned” crocodile, *Voay robustus*, collected from Holocene deposits in southwestern Madagascar. Phylogenetic analyses of partial mt genomes and tip-dated timetrees based on molecular, fossil, and stratigraphic data favor a sister group relationship between *Voay* and *Crocodylus* (true crocodiles). These well supported trees conflict with recent morphological systematic work that has consistently placed *Voay* within Osteolaeminae (dwarf crocodiles and kin) and provide evidence for likely homoplasy in crocodylian cranial anatomy and snout shape. The close relationship between *Voay* and *Crocodylus* lends additional context for understanding the biogeographic origins of these genera and refines competing hypotheses for the recent extinction of *Voay* from Madagascar.

## Abstract (web summary)

Hekkala et al. use mitochondrial genomic data of the extinct “horned” crocodile, *Voay robustus*, from Holocene deposits in Madagascar to examine its evolutionary history. Phylogenetic analyses indicate a sister group relationship between *Voay* and true crocodiles (*Crocodylus*), and add support to an African origin for the group.

## Introduction

New methods to recover genomic data from extinct lineages have helped to clarify previously enigmatic phylogenetic relationships and enabled rigorous tests of biogeographic and evolutionary hypotheses^1–4^. In some cases, molecular data from extinct Holocene island faunas have revealed surprising biogeographic scenarios^5–8^. Additional ancient DNA studies, including recent analyses of our own family, Hominidae^9,10^, canids^11^ and elephants^12^ have yielded dramatic insights into the role of admixture between divergent lineages in evolutionary history. Genomic data for extinct species could also yield insights into extinction mechanisms that operated in the recent past^13,14^.

**Fig. 1:**
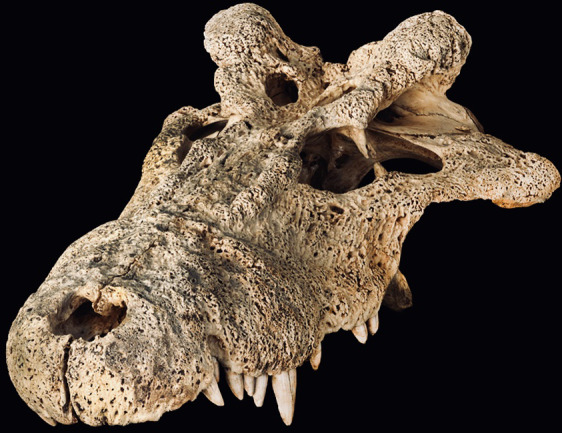
Subfossil skull of *Voay robustus* (AMNH FR-3102) from southwestern Madagascar. A skull of *Voay robustus* collected at Ampoza (44° 42.3’ E, 22° 18.9’ S, 570 m elevation) during the joint Mission Franco-Anglo-American expedition from 1927–1930 (White, 1930).

The arrival of modern humans in Madagascar between ~9000 and ~2500 YBP^15–20^ preceded the extinction of much of the island’s vertebrate megafauna including giant tortoises (*Aldabrachelys* spp.), elephant birds that ranged to enormous size (*Aepyornis*, *Mullerornis*, *Vorombe*), dwarf hippos (*Hippopotamus lemerlei, H. madagascariensis*), and several lemur species (*Megaladapi*s, *Archaeoindris, Palaeopropithecus, Pachylemur*)^6,13,21–23^. One lesser-known extinction that occurred during this period was the demise of an endemic “horned” crocodile, *Voay robustus* (Fig. 1). Early explorers to Madagascar noted that Malagasy peoples consistently referred to two types of extant crocodiles on the island, a large robust crocodile and a more gracile form with a preference for rivers^24^. This suggests that both types persisted until very recently^24,25^, but only the gracile form, now recognized as an isolated population of the Nile crocodile (*Crocodylus niloticus*), currently is found on the island^26^.

Despite nearly 150 years of investigation, the phylogenetic position of the extinct horned crocodile of Madagascar remains controversial. In 1872, the earliest description of the species by Grandidier and Vaillant^27^ noted differences between sub-fossil cranial and postcranial material excavated from Holocene deposits near Amboulisatre and extant crocodiles (*C. niloticus*) in Madagascar. Based on the robustness of available skeletal features, including vertebral, dental and cranial elements and snout shape, (Fig. 1), Grandidier and Vaillant named the extinct form *Crocodylus robustus*^27^. They suggested a possible affinity between the subfossil material and *Crocodylus niger*, now recognized as the dwarf crocodile (*Osteolaemus tetraspis*) that is native to west-central Africa. In the same year, Grandidier^28^ further contrasted the relatively stout features of *C. robustus* with those of the more gracile *C. niloticus* that currently inhabits the island. Barbour^29^ and Boettger^30^ however, suggested that the extinct robust species simply represented an aged *C. niloticus*. In 1910, Vaillant and Grandidier^24^ and later Mook^31,32^ examined subfossil material from additional sites and upheld *C. robustus* as clearly distinct from extant *C. niloticus*. Mook^32^ noted that *C. robustus* was instead more similar to the extant saltwater crocodile, *C. porosus*.

**Fig. 2:**
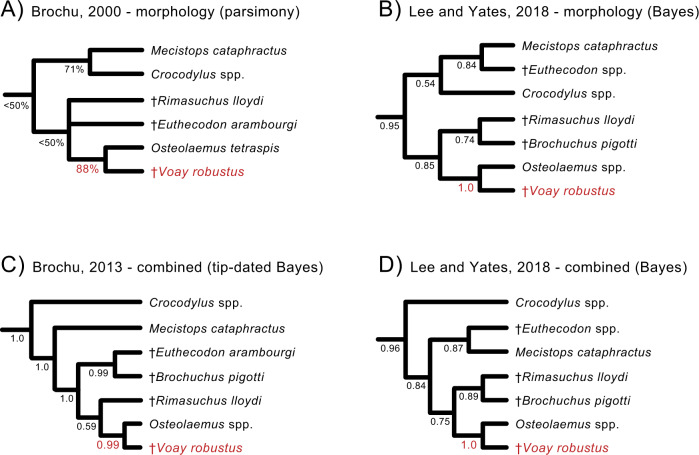
Prior cladistic and Bayesian analyses supporting a grouping of *Voay robustus* (red) with the genus *Osteolaemus* (dwarf crocodiles). Four representative phylogenetic hypotheses are shown (**A**–**D**). Both morphology (**A**, **B**) and combined analyses of morphology plus molecules (**C**, **D**) place *Voay* with *Osteolaemus* and extinct African osteolaemines to the exclusion of *Crocodylus* (true crocodiles). The tip-dated tree in (**C**) is from a Bayesian reanalysis of morphological data from Brochu (2013) in combination with DNA sequence data (Lee and Yates, 2018). Support scores at nodes are parsimony bootstrap percentages (**A**) or Bayesian posterior probabilities (**B**–**D**). Robust support for *Voay* + *Osteolaemus* is highlighted in red.

After conducting a detailed morphological study of available subfossil material representing *C. robustus*, Brochu^33^ noted that *C. robustus* lacks many of the distinguishing features of the genus *Crocodylus*. In his detailed cladistic analysis, the extinct Malagasy species grouped with extant dwarf crocodiles (*Osteolaemus* spp.)^33^ of west and central Africa. Several phenotypic characters allied *C. robustus* with the genus *Osteolaemus*, some of which might relate to overall skull shape with a relatively short and deep snout in both taxa. Based on this evidence, Brochu erected a new monotypic genus, *Voay* (the modern Malagasy word for extant crocodiles) within Osteolaeminae, resulting in the current species name *Voay robustus*^33^. Subsequent phylogenetic analyses of morphology^34–39^ as well as total evidence analyses of morphology and molecules^37,39–41^ have consistently clustered *V. robustus* and *Osteolaemus* to the exclusion of other crocodylian genera, with *Crocodylus* distantly related to *Voay* (Fig. 2).

Here, we use mitochondrial (mt) capture and whole genome enrichment (WGE) of ancient DNA (aDNA) to recover mt sequences from two subfossil specimens of *Voay robustus*. We employ separate and combined analyses of mtDNA and morphological data to test competing hypotheses for the phylogenetic placement of *Voay* relative to living and extinct crocodylians. Total evidence analyses that merge molecular, fossil, and stratigraphic data yield timetrees for Crocodylidae that we utilize to better characterize the biogeographic history of the clade, the timing of *Crocodylus* origins, and the extinction of *Voay*.

## Results

### Carbon dating

*Voay* sample AMNH FR-3101 yielded AMS ^14^C dates of 1450 ± 30 (1422–1307) ^14C^ yr BP, while AMNH FR-3103 yielded dates of 1380 ± 30 (1364–1280) ^14C^ yr BP. Newly derived ^14^C dates are slightly younger than those recovered from vertebrates from the same deposits ca. 1,800 ^14^C yr BP and 2,430 ^14^C yr BP^42^, confirming the relatively recent age of the specimens.

### Recovery of partial mt genomes for *Voay robustus*

Whole genome enrichment (WGE) and targeted mt capture approaches yielded partial mt genomes from two Holocene specimens of *Voay robustus* - AMNH FR-3101 and AMNH FR-3103 (Supplementary Data 1). WGE produced both mtDNA and nuclear sequences from *Voay* samples, but coverage for single-copy nuclear genes was low. We therefore focused phylogenetic analyses on mt reads derived from both enrichment procedures. Authenticity of the mtDNA data for *Voay* was evidenced by DNA-damage patterns, low sequence divergence between individuals, clean negative controls, and consistent phylogenetic placement of sequences from replicated processing of both specimens (Supplementary Figs. 1–2; Supplementary Table 1 and Data 3; Supplementary Data 2).

Four reconstructions of the *Voay* mt genome were assembled by mapping short reads from these two specimens to two reference mt genomes (*Osteolaemus* and *C. porosus*) using the EAGER pipeline (see Materials and Methods). The most complete reconstruction, *Voay* AMNH FR-3101 *C. porosus* ref., had 18% missing data relative to the reference genome sequence for mt ribosomal DNAs (rDNAs) and protein-coding genes. Reconstructions using *Osteolaemus* as the reference and/or *Voay* specimen AMNH FR-3103 yielded mt datasets with more missing data relative to reference genomes (45–72% missing). Average sequencing coverage for the four mt genome builds was as follows: *Voay* AMNH FR-3101 *Osteolaemus* ref. (5X); *Voay* AMNH FR-3103 *Osteolaemus* ref. (3X); *Voay* AMNH FR-3101 *C. porosus* ref. (5X); *Voay* AMNH FR-3103 *C. porosus* ref. (4X). Pairwise comparisons among the four *Voay* builds from two different specimens show minor divergence from each other at the nucleotide level. Short reads for each *Voay* specimen were deposited at NCBI (short read archive Bioproject PRJNA681754).

### Phylogenetic analyses and evolutionary inferences

**Fig. 3:**
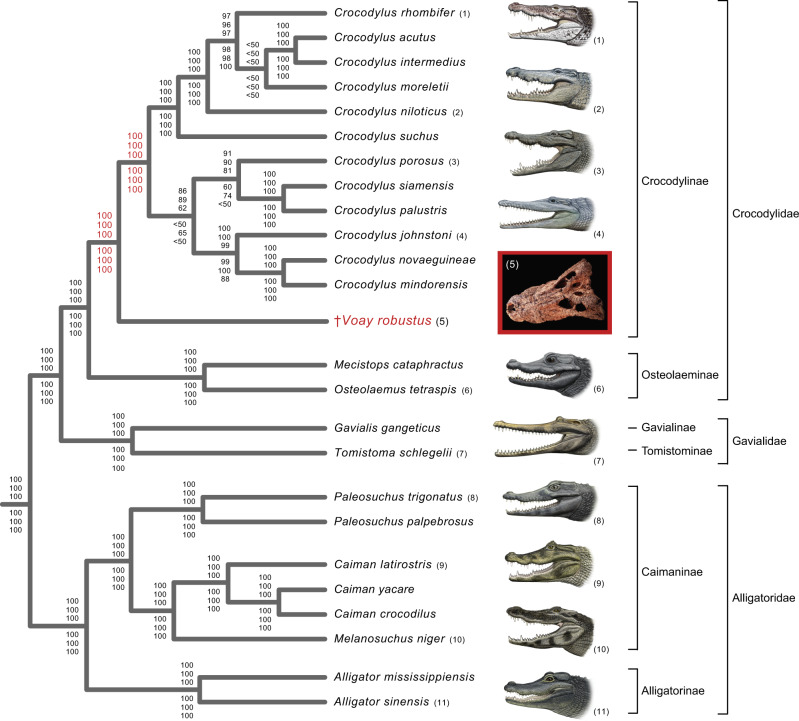
Phylogenetic relationships of *Voay robustus* based on partial mitochondrial (mt) genomes support a sister group relationship between *Voay* and a monophyletic *Crocodylus* (true crocodiles). The tree shown is based on ML analysis (partitioned by gene) and includes data from all four builds of the *Voay* mt genome. Bootstrap scores at each node are (from top to bottom): all four builds of *Voay* mt genome with partitioned ML analysis, *Voay* AMNH FR-3101 *C. porosus* reference build with partitioned ML analysis, *Voay* AMNH FR-3101 *Osteolaemus* reference build with partitioned ML analysis, *Voay* AMNH FR-3103 *C. porosus* reference build with partitioned ML analysis, *Voay* AMNH FR-3103 *Osteolaemus* reference build with partitioned ML analysis, and all four builds of *Voay* mt genome with equally-weighted parsimony analysis. Bootstrap scores for the two internodes that bound the branching point of *Voay* are highlighted in red. All trees were rooted with bird, turtle, and lizard outgroups (not shown). Higher level taxa are delimited by brackets to the right of species names. Paintings of crocodylians are by C. Buell, and photo of *Voay* (AMNH FR-3101) is by E. Hekkala.

All phylogenetic analyses of our mtDNA datasets (Fig. 3; Supplementary Table 1 and Data 3; Supplementary Data 2) corroborate relationships among the nine extant genera of Crocodylia that have been consistently supported by molecular data since 2008^43^. The genus *Crocodylus* (true crocodiles) is sister to a clade composed of *Mecistops* (African slender-snouted crocodiles) and *Osteolaemus* (dwarf crocodiles) within Crocodylidae (Fig. 3). Crocodylidae groups with Gavialidae (true and false gavials), and this combined clade is sister to Alligatoridae (alligators and caimans). However, our mt trees contradict previous numerical phylogenetic analyses of morphology and combined data that robustly cluster *Voay* with osteolaemines (Fig. 2^33–41,44^). Our mtDNA trees instead reflect a closer association with *Crocodylus* as hypothesized by earlier authors^29,30,32,45^ (Fig. 3). Parsimony and maximum likelihood (ML) analyses of partial mt genomes (two rDNAs and 13 protein-coding genes) uniformly support a sister group relationship between *Voay* and a monophyletic *Crocodylus*, as well as a clade composed of *Osteolaemus* and *Mecistops* (Fig. 3). These relationships are robustly supported by all 64 analyses of the molecular dataset (Supplementary Table 1 and Data 3; Supplementary Data 2). ML phylograms show that *Voay* branches from the stem lineage of extant *Crocodylus* at about the midpoint of this long internal branch, with limited divergence among the four partial mt genome builds reconstructed from the two *Voay* specimens (Supplementary Fig. 2).

**Fig. 4:**
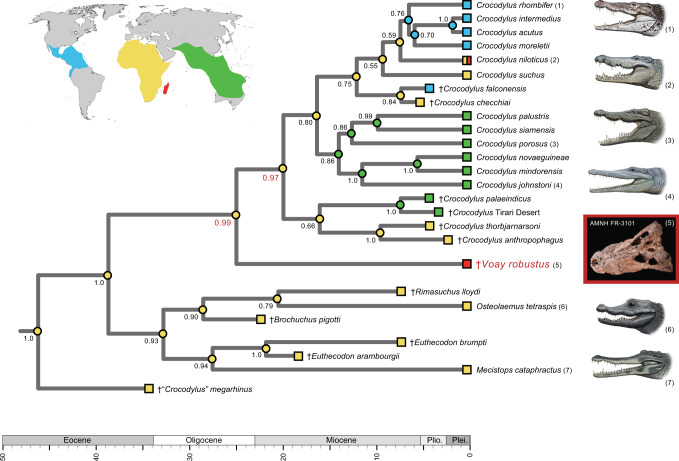
Tip-dated Bayesian timetree showing the phylogenetic relationships of *Voay robustus* relative to extant and extinct crocodylids with a mapping of geographic distributions (colored squares at tips of branches). Bayesian posterior probabilities are at nodes; support scores for the two internodes that bound the branching point of *Voay* are highlighted (red). Optimization of geographic regions to internal nodes (colored circles) is based on equally-weighted parsimony and implies an African ancestry for the overall clade with minimally two migrations to Australia/Asia, two to the New World, and two to Madagascar. An identical mapping of ancestral areas results for minimum area change (MAC) parsimony analysis. The *Voay* AMNH FR-3101 *C. porosus* mt genome build (partitioned by 1^st^, 2^nd^, 3^rd^ codons) was employed in combination with morphological characters and stratigraphic data from Lee and Yates (2018). Taxa that are distantly related to *Voay* are pruned from the figure; for the complete timetree, see Supplementary Data 2. Paintings of crocodylians are by C. Buell; photo of *Voay* (AMNH FR-3101) is by E. Hekkala.

In all six tip-dated timetrees (Supplementary Table 1 and Data 3; Supplementary Data 2), *Voay* again groups with *Crocodylus*, and *Osteolaemus* clusters with *Mecistops* (Fig. 4). Some extinct taxa that were coded for just phenotypic characters are unstable in these combined data analyses, but for each Bayesian tip-dating tree, the relationships of *Voay* to extant crocodylid genera are consistent in 100% of the trees in posterior distributions and congruent with all 64 analyses of mtDNA alone (Fig. 3; Supplementary Data 2). In terms of topology and divergence times, our most complete timetree for Crocodylia (Fig. 4) closely matches the hypothesis proposed by Lee and Yates^37^, except for the conflicting position of *Voay* relative to *Crocodylus*, *Osteolaemus*, and *Mecistops*. Our tip-dated tree shows a long unbranching lineage that split from all other sampled crocodylians in the late Oligocene and terminates at the Holocene extinction of *Voay* on Madagascar. This divergence from the genus *Crocodylus* dates to ~24.9 Ma (95% highest posterior density [HPD] = 18.8–32.1 Ma), with the earliest split among *Crocodylus* species (crown + stem) dated at ~19.9 Ma (95% HPD = 14.7–26.2 Ma) and ~16.3 Ma for crown group *Crocodylus* (95% HPD = 12.5–20.5 Ma). *Voay* separated from the more distantly related *Osteolaemus* in the Eocene at ~38.6 Ma (95% HPD = 32.4–45.3 Ma) (Fig. 4). By contrast, *Voay* split from *Osteolaemus* just ~17.8 Ma in the combined data timetree of Lee and Yates^37^ and at ~16.4 Ma in their tip-dated analysis of morphological characters. Our six timetree hypotheses show some variation in median divergence time estimates, due to differences in character coding, ordering of character states, and taxon sampling in the two morphological datasets that were reanalyzed^37,46^, as well as the inclusion or exclusion of 3^rd^ codon positions from mt protein-coding genes (Supplementary Data 2). For example, across our six tip-dated trees, the median divergence date for *Crocodylus* and *Voay* ranges from ~22.1–27.7 Ma, and the split between *Voay* + *Crocodylus* and *Osteolaemus* + *Mecistops* ranges from ~30.7–38.6 Ma. However, we suggest caution when interpreting these dates due to disagreements on specimen dating in the published literature, and the possibility that errors in published dates may affect results of these analyses.

Parsimony optimizations of geographic ranges on our tip-dated timetrees consistently reconstruct an African ancestry for both *Voay* and *Crocodylus* (Fig. 4). Although alternative reconstructions are nearly as parsimonious, the ‘out of Africa’^33^ pattern generally holds for our maximum clade credibility (MCC) timetrees. A migration of the ancestral *Voay* lineage from Africa to Madagascar is inferred, but this biogeographic shift is not well-constrained temporally given the current sampling of extinct taxa.

Mapping of morphological characters on our most complete tip-dated tree (Fig. 4) implies convergent homoplasy in multiple characters that instead group *Voay* with *Osteolaemus* in previous phylogenetic analyses of morphology and combined data (Fig. 2). For the tip-dated timetree of Lee and Yates^37^ that did not include any molecular data for *Voay*, seven morphological characters are synapomorphic for a *Voay* + *Osteolaemus* clade. All seven of these cranial characters are interpreted as convergences on our tip-dated tree (Supplementary Table 1 and Data 3). Prominent squamosal “horns”, as seen in *Voay* (Fig. 1), also evolved convergently in four additional crocodylid taxa (*Crocodylus rhombifer*, *C. siamensis*, *C. anthropophagus* + *C. thorbjarnarsoni*, and *Euthecodon brumpti*). For the taxa sampled here, this trait is restricted to just Crocodylidae but is highly homoplastic (consistency index = 0.200). There is limited morphological support, just one unambiguously optimized synapomorphy, for the novel grouping of *Voay* sister to *Crocodylus* in our tip-dated tree. Transformation from a straight or gently curved prefrontal-frontal suture to an ‘L’-shaped suture optimizes to the common ancestor of *Voay* + *Crocodylus* (Supplementary Table 1 and Data 3). This labile binary character shows minimally 11 changes on the overall tree (consistency index = 0.091).

## Discussion

Our combined WGE and targeted mtDNA capture recovered partial mt genomes for two Holocene specimens of *Voay robustus* from Madagascar (Supplementary Data 1) and enabled the first molecular phylogenetic placement of this extinct island endemic. Molecular and combined data uniformly position *Voay* as sister to *Crocodylus* and outside of the clade comprised of *Osteolaemus* and *Mecistops* (Figs. 3, 4; Supplementary Data 2). All of our trees contradict previous quantitative phylogenetic work that consistently placed *Voay* within Osteolaeminae, close to the genus *Osteolaemus* (dwarf crocodiles) based on anatomical characters and combined analyses of morphology plus molecules (Fig. 2^33–41,44^).

For Crocodylia, prior molecular phylogenetic work suggested that morphological features are commonly characterized by high levels of homoplasy^40,47,48^ that may be driven by convergent ecological and functional pressures^49–57^. Morphological data provide minimal character support for grouping *Voay* as the sister group to *Crocodylus*. Just one morphological character change unequivocally maps to the last common ancestor of the clade, and this grouping implies convergent homoplasy in a host of anatomical features shared by *Voay* and *Osteolaemus* (Supplementary Table 1 and Data 3). Our results further highlight ongoing conflicts between morphological and molecular characters in crocodylian phylogenetics with the caveat that the mt genome is generally interpreted as a single non-recombining locus in Crocodylia^43^. Corroboration from independent nuclear loci would solidify support for our novel phylogenetic hypotheses (Figs. 3, 4).

Recent molecular hypotheses of crown group relationships within Crocodylidae have been equivocal for interpreting both the age and biogeographic origins of the genus *Crocodylus*^58–60^. The “out of Africa”^33^ hypothesis for crown group *Crocodylus* was tested in a probabilistic framework by Oaks who found stronger support for origin of the group in Australia/Asia^58^. The mt genome analysis of Meredith et al. supported monophyly as opposed to paraphyly of *Crocodylus* spp. from Australia and Asia^59^. For their tree, parsimony optimization of geography implies an African origin for *Crocodylus*, with a recent dispersal from Africa to the New World and another dispersal to Australia/Asia, which agrees with earlier morphological work. More recently, Nicolaï and Matzke^61^ partitioned geographic areas more finely and reconstructed an Asian origin for *Crocodylus*. Like Oaks and Meredith et al., this study did not directly consider the extensive fossil diversity of *Crocodylus* and more generally, Crocodylidae (e.g., ^33–36^).

Our biogeographic reconstructions that include fossils instead suggest an African origin for *Crocodylus*. In tip-dated MCC timetrees, extinct taxa that are closely related to *Crocodylus* (*Rimasuchus, Brochuchus*, *Euthecodon*, *“Crocodylus” megarhinus*) are predominantly African, as are extant outgroup taxa, *Osteolaemus* and *Mecistops* (e.g., Fig. 4). Taken together with the placement of *Voay* from Madagascar as the sister taxon to *Crocodylus*, our timetrees hint at an African origin for the genus^62^. However the unstable affinities of various extinct *Crocodylus* spp. in our timetrees complicate interpretation and beg for more comprehensive analyses in the future that incorporate the full complement of extinct geographic diversity and a broader survey of informative characters. Crown group *Crocodylus* initially diversified at ~16.3–17.7 Ma according to our four tip-dated MCC timetrees that sample all extant species in the genus. However, in these, as in other analyses^58,60^, broad 95% HPDs limit interpretation (e.g., 12.5–20.5 Ma for the *Voay* AMNH FR-3101 *C. porosus* ref. alignment).

The inferred migration or vicariance event that isolated the *Voay* evolutionary lineage on Madagascar is not well-constrained according to tip-dated timetrees. In our most comprehensive hypothesis (Fig. 4), *Voay* diverged from its sistergroup, *Crocodylus*, at ~24.9 Ma (95% HPD = 18.8–32.1 Ma), and there is no evidence for speciation or extinction in the *V. robustus* lineage up until the final demise of this single species in historical times. Movement of *Voay* to Madagascar therefore may have occurred between the late Oligocene and the first known occurrences of *Voay* in the Pleistocene, ~10,000 years ago^42,63^. Over this time span, Madagascar was fully isolated from Africa and other continental landmasses, so any dispersals were necessarily trans-oceanic^13,64,65^. Recent prevailing winds and ocean currents oppose overwater dispersal from Africa to Madagascar due to north or south-southwest flow, but some paleo-oceanographic models reconstruct intermittent and rare eastward flow in the Eocene and Oligocene^66^. The salt tolerance of extant *Crocodylus* spp. has been suggested as a driver for the relatively recent range expansion of this genus^59,61^, but the reconstruction of salt tolerance in extinct species, such as *Voay*, is ambiguous given the distribution of this trait in extant crocodylians^67–71^. Overall, our phylogenetic hypotheses (Fig. 4) broadly delimit the timing of biogeographic events, but future paleontological discoveries, in particular extinct taxa that branched from the long ~24.9 MY *Voay* lineage, are required to further refine this timeframe.

Several explanations have been proposed for the extinction of megafauna in Madagascar during the transition to the Anthropocene^17,72–74^. Currently viable hypotheses include environmental change or over-exploitation and habitat alteration by humans that together may have acted as synergistic drivers of megafaunal collapse across the island^17,18^. Bickelmann and Klein^75^ argued that, given the absence of evidence for direct human exploitation, competition with the Nile crocodile, *C. niloticus*, was the more likely driver of *Voay*’s extinction. Closely related species of similar body size often share similar ecologies^76–79^, and the molecular evidence for a more recent common ancestry with *Crocodylus* spp. (Figs. 3, 4) relative to the previous consensus that grouped *Voay* with *Osteolaemus* (Fig. 2) perhaps lends additional support to the competition hypothesis. Relaxed clock estimates of the Nile crocodile’s arrival in Madagascar suggest a very recent invasion ca. 2,000–3,000 YBP^60^ that implies temporal overlap with *Voay*, but the earliest documented Nile crocodile material in Madagascar dates to just ~310–460 years ago^80^.

A more speculative extinction scenario also requires a temporal and geographic overlap between *Voay* and *C. niloticus* in Madagascar. The mixing of genes between differentiated evolutionary lineages (‘phylogenetic species’) is well documented between some species in the genus *Crocodylus*^81^. It is therefore at least possible that introgressive hybridization with the recently invading *C. niloticus* contributed to decline of *Voay* through genetic swamping or ‘extinction via hybridization’^82^. Ancient mtDNA sequences from *Voay*, however, do not provide any compelling evidence for hybridization with the Nile crocodile. Recent introgression of mtDNA would be expressed as a clustering of these two species in mt trees as has been found in *C. acutus* and *C. rhombifer*^83,84^, which is not supported (Fig. 3). Future ancient DNA work that focuses on recovery of *Voay* nuclear DNA promises a more rigorous test of gene flow hypotheses.

Given the concurrent extinction of megafauna on Madagascar, it is perhaps more plausible that *Voay* succumbed to a combination of direct extirpation by humans and rapid environmental change^33^. Unlike large mammalian taxa such as hippos and lemurs, that were likely targeted as adults by humans, *Voay* populations may have been impacted by exploitation of eggs, resulting in a rapid decline. Vaillant and Grandidier noted that both species of crocodiles were recognized by communities throughout Madagascar and that crocodile eggs were regularly consumed, particularly in southwestern Madagascar^24^. This type of impact would be largely undetectable at archeological sites through modern taphonomic measures.

Our study provides the first molecular systematic characterization of *V. robustus* and indicates that this recently extinct island endemic represents the sister lineage to *Crocodylus* (true crocodiles). Molecule-based trees (Fig. 3) and combined phylogenetic analyses of molecules and morphology (Fig. 4) contradict trees from previous studies that grouped this species and dwarf crocodiles (*Osteolaemus*) with high support (Fig. 2). Tip-dated timetrees suggest that *Voay* diverged from *Crocodylus* near the Oligocene/Miocene boundary (~22.1–27.7 Ma) and represents a relict lineage that survived to historical times in Madagascar but has no known close relatives, living or extinct (Fig. 4; Supplementary Data 2). Our results highlight the value of ancient DNA for uncovering novel, unexpected evolutionary relationships and providing context for new interpretations of morphological evolution, biogeographic history, and extinction patterns.

## Methods

### Specimens and sample processing

The paleontological collections at the American Museum of Natural History (AMNH) include a series of specimens of *Voay* (= *Crocodylus*) *robustus* from Ampoza, Madagascar (44° 42.3’ E, 22° 18.9’ S, 570 m elevation). These specimens were collected during the joint Mission Franco-Anglo-American expedition from 1927–1930^85^. White’s descriptions of field excavations denote a Holocene deposition^85^, and subsequent C_14_ dating of adjacent faunal remains from Ampoza are dated from ~2500–1000 YBP^63^. Interpretation of specific depositional context of *V. robustus* material is limited. However, White’s notes and photographs from the excavation indicate a solid surface layer of limestone, below which a dark soil held diverse disarticulated skeletal elements^85^. As excavations proceeded, the site filled with water from subsurface layers, and field laborers extracted material from underwater^85^. A reconstruction of the habitat suggests a riparian stream system near a marsh^86^.

Two specimens were targeted as potential sources of ancient DNA. The sampling plan was designed to minimize damage to the specimens and reduce contamination. Prior to handling specimens, all tools were sterilized by UV radiation for 15 min, soaked in DNAaway (Thermo Scientific) for 5 min, and then dried in a covered sterile chamber. For each skull, a tooth was gently lifted to expose an un-erupted tooth beneath. One un-erupted tooth from each specimen was removed for genomic analysis. All surfaces of tooth samples were rinsed with 70% DNAaway for 30 s, rinsed twice with sterile water, and then dried in a covered petri dish. Each tooth was subsequently placed in a sterile 15 ml falcon tube. Parallel sample processing and negative controls were executed during the specimen sampling and all subsequent DNA extraction processes (Supplementary Fig. 1).

### Carbon dating

Samples from each specimen (AMNH FR-3101 and AMNH FR-3103) were sent to Beta Analytic Inc, Miami Florida for radiocarbon dating. Teeth were initially decalcified and gelatinized using EDTA and HCl. Once collagen preservation was confirmed, samples were radiocarbon dated and calibrated dates reported. Calibration was calculated using one of the databases associated with the 2013 INTCAL program. Conventional Radiocarbon Ages and Sigmas are rounded to the nearest 10 years per the conventions of the 1977 International Radiocarbon Conference. When counting statistics produce Sigmas lower than ±30 years, a conservative ±30 BP is cited for the result. All work was performed under strict chain of custody and quality control under ISO/IEC 17025:2005 Testing Accreditation PJLA #59423 accreditation protocols. Sample, modern and blanks were all analyzed in the same chemistry lines by qualified professional technicians using identical reagents and counting parameters within on Beta Analytic Inc’s own particle accelerators.

### DNA isolation

Subsampling of the two tooth specimens (AMNH FR-3101 and AMNH FR-3103) was done at the AMNH, and duplicate samples were shipped to the University of British Columbia (UBC). Isolation of ancient DNA was replicated in dedicated clean room facilities at the AMNH and at UBC (Supplementary Fig. 1) according to published protocols^87^. For the ancient DNA extractions conducted at the AMNH, between 50 and 90 mg of surface sterilized tooth was crushed and demineralized overnight at room temperature in 1 mL 0.5 M EDTA with gentle shaking. Samples were then digested in 750 µL of a sarcosyl-based proteinase K solution and purified using the MinElute PCR Purification kit (Qiagen) with two washes of 700 µL Buffer PE and eluted twice in 80 µL (2 × 40 µL) buffer EB at 0.05% Tween-20. For the ancient DNA extractions conducted at UBC, a modified version of extraction protocol Y was employed, as originally described by Gamba et al.^87^. Each sample was extracted in duplicate at UBC. Approximately 250 mg of each sample was ground while submerged in liquid nitrogen using a Spex 6770 freezer mill (5 min precooling, 1 min of grinding at 10x per second). Samples were demineralized in 3 mL of 0.5 M EDTA pH 8.0, 150 μL 10% SDS, and 100 μL of 20 mg/ml Proteinase K, with incubation overnight at 56 °C. The lysate was concentrated to 250 μL using Amicon Ultra-4 30 kDa tubes by centrifugation. The resulting 250 μL of lysate were mixed with 5x volume of buffer PB and added in three steps to a MinElute (Qiagen) column and centrifuged, removing the flow-through after each step. The column was washed twice with 750 μL of PE and centrifuged, allowing desalting for 5 min during the first wash. The elution was performed using 50 μL of ultra-pure water preheated to 56 °C.

Genomic DNA replicates from both laboratories were shipped on dry ice to Arbor Biosciences, (Ann Arbor, Michigan, USA) for subsequent library preparation and enrichment processing.

### Library preparation

Two duplicate Illumina® libraries for each *Voay* specimen were prepared in ancient DNA processing facilities by Arbor Biosciences for use in downstream WGE and targeted sequence capture of mtDNA. Each library was amplified using unique P5 and P7 indexing primers, and 10 µL of each library in 40 µL reactions were quantified on a CFX96 Real-time PCR machine (BioRad). Indexed libraries were purified using MinElute (Qiagen) columns.

### Whole genome enrichment (WGE) using RNA baits

We enriched for crocodylian genomes using a modified protocol wherein genomic DNA (gDNA) from closely related taxa are converted into biotinylated RNA baits^3,88^. Briefly, at the AMNH, gDNA was extracted from ten modern crocodylian blood samples representing six taxa [*Crocodylus moreletii* (*n* = 1), *C. acutus* (*n* = 1), *C. siamensis* (*n* = 1), *C. suchus* (*n* = 2), *C. niloticus* (*n* = 2) and *Osteolaemus* tetraspis (*n* = 3)] using a Qiagen DNeasy kit and the manufacturer’s protocols for nucleated red blood cells. Approximately 1 µg of extracted DNA from each species was sent to Arbor Biosciences (Ann Arbor, Michigan USA) for global reverse transcription (both strands) with biotinylated rUTP using their proprietary procedure^3^. This yielded an aqueous suspension of approximately 100 µg of mixed crocodylian RNA baits for subsequent WGE.

Enrichment of *Voay robustus* genomic libraries was conducted at Arbor Biosciences according to their MYcroarray capture protocol version 3 (https:// arborbiosci.com/wp-content/uploads/2017/10/MYbaits-manual-v3.pdf). Briefly, each capture reaction used 1 µg of crocodylian RNA baits, 9 µL-indexed library (described above), and the MYBaits (MYcroarray) kit protocol for enrichment. Hybridizations were done at 48 °C for 48 h. Following SPRI bead cleanup and MinElute purification, enriched eluates were amplified for 10 cycles and then again purified with MinElute columns. Approximately 9 µL of these purified products were used in another round of capture using identical conditions as the first round, except incubation occurred at 55 °C for 39 h. Reactions were again bead-cleaned and purified with MinElute columns. Purified products were then re-amplified for 5 cycles and the resulting re-amplified, doubly-enriched libraries were purified one last time using MinElute columns.

### Targeted mtDNA enrichment using synthetic baits

A previously developed MYbaits kit that targets the crocodylian mt genome was used for enrichment of the ancient crocodylian DNA libraries. Each capture reaction used 1 µg of crocodylian mt capture baits, 9 µL-indexed library (described above), and the MYBaits kit protocol version 3 (described above) for enrichment. Hybridizations were done at 48 °C for 48 h. Following bead cleanup and MinElute purification, enriched eluates were amplified for 10 cycles and then purified with MinElute columns. Purified products were then re-amplified for 5 cycles and the resulting re-amplified, doubly-enriched libraries were purified one last time using MinElute columns.

### DNA sequencing

For each of the two *Voay robustus* specimens (AMNH FR-3101 and AMNH FR-3103), two independent samples plus negative controls were extracted (A and B), two replicate libraries were produced (1 and 2) and one pooled WGE and Mito enriched library were sequenced, resulting in 10 separately processed samples. For each specimen replicate set (either 3101 or 3103), the indexed whole genome enriched library and the targeted mtDNA enriched library were pooled with a ratio of 75 (WGE library)/25 (mtDNA capture library), and sequenced using one full lane on an Illumina HiSeq® 2500 (paired-end, 150 bp reads) at the New York Genome Center (see Supplementary Figure 1 for sample AMNH FR-3101example).

### Sequence analyses and mtDNA reconstruction

Preliminary mapping analyses using EAGER, an ancient genomics pipeline^89^, showed that crocodylian mtDNA was not recovered from the negative control libraries. Exploratory mapping of short reads also indicated that mtDNA builds derived from AMNH and UBC libraries were homogeneous for each *Voay* specimen and that libraries derived from the same specimen could be safely combined for final reconstruction of ancient mt genome sequences. Merged sequence reads from *Voay* AMNH FR-3101 and merged reads from *Voay* AMNH FR-3103 were analyzed separately using EAGER, which automates read processing, mapping, variant detection, and consensus genome reconstruction. Mapping against a crocodylian reference genome enables screening of non-endogenous DNA from the often complex metagenomic mixtures in ancient samples. Moreover, these reference alignments highlight erroneous base incorporations that can signify DNA damage that is a characteristic of ancient samples^90^.

Using the EAGER pipeline, reads were processed by clipping adapters, merging paired ends with overlapping regions, and trimming bases with phred scores lower than 20. So that the reconstructed ancient mt genomes would not be biased toward one or the other genus that were a priori hypothesized to be closely related to *Voay* (ref. ^91^), merged reads were mapped to both *Crocodylus porosus* (GenBank accession # DQ273698.1) and *Osteolaemus tetraspis* (GenBank accession # NC_009728) reference mt genomes. Merged reads of minimum length 30 were treated as single-end and aligned to the reference genomes using BWA-MEM and default settings. After removing duplicates, the UnifiedGenotyper module in the Genome Analysis Toolkit (GATK) was used to make variant and reference base calls at each position. Both variant and reference calls were required to have the support of at least two reads, a phred-scaled genotype quality score of at least 30, and a consensus SNP frequency of at least 90%. Failing these criteria at any given position in the reference resulted in the insertion of an ‘N’ ambiguity character. With alleles compiled, EAGER’s VCF2Genome module was used to generate draft genome sequences relative to the *C. porosus* and *Osteolaemus* references. To verify the reconstruction of the ancient mt genome, EAGER’s DamageProfiler module was used to quantify alignment errors resulting from ancient DNA damage. A separate mitogenomic reconstruction for each specimen relative to each reference genome resulted in four total *Voay* mt genome sequences (*Voay* AMNH FR-3101 *Osteolaemus* ref.; *Voay* AMNH FR-3103 *Osteolaemus* ref.; *Voay* AMNH FR-3101 *C. porosus* ref.; Voay AMNH FR-3103 *C*. porosus ref.) for use in downstream analyses.

### Phylogenetic methods

The molecular dataset included the new *Voay robustus* mt genome reconstructions and previously published mt genomes from 22 extant species of Crocodylia including [Genbank # in brackets]: *Alligator mississippiensis* [NC_001922]*, Alligator sinensis* [NC_004448]*, Caiman crocodilus* [NC_002744]*, Paleosuchus palpebrosus* [NC_009729]*, Paleosuchus trigonatus* [NC_009732]*, Gavialis gangeticus* [NC_008241]*, Tomistoma schlegelii* [NC_011074]*, Mecistops cataphractus* [NC_010639]*, Osteolaemus tetraspis* [NC_009728]*, Crocodylus acutus* [NC_015647]*, Crocodylus intermedius* [JF502242]*, Crocodylus johnstoni* [NC_015238]*, Crocodylus mindorensis* [NC_014670]*, Crocodylus moreletii* [NC_015235]*, Crocodylus suchus* [JF502244]*, Crocodylus niloticus* [JF502246]*, Crocodylus novaeguineae* [JF502240]*, Crocodylus palustris* [NC_014706]*, Crocodylus porosus* [DQ273698]*, Crocodylus rhombifer* [JF502247], and *Crocodylus siamensis* [EF581859]. We also included three newly generated partial mt genomes from *Caiman yacare* ([MN885913] sample ID# C058), *Caiman latirostris* ([MN885912] sample ID# S234), and *Melanosuchus niger* ([MN885911] sample ID# 92042). Blood samples were provided by St. Augustine Alligator Farm Zoological Park (St. Augustine, Florida, USA), and protocols for DNA extraction, PCR amplification, and sequencing are outlined in Meredith et al.^59^. Combined, this set of taxa includes *Voay* and most currently recognized extant crocodylian species with the exception of recent splittings of *Mecistops* and *Osteolaemus* into multiple phylogenetic species^92–94^. One representative mt genome was included from each of these two genera (see above).

Sauropsid mt genomes were included as outgroups to root mtDNA trees. Aves generally is considered the extant sister group to Crocodylia, with Lepidosauria and Chelonia being more distantly related within the clade Sauropsida^95,96^. Outgroups for our phylogenetic analyses included one lizard (*Anolis carolinensis* [NC_010972]), two turtles (*Pelodiscus_sinensis* [AY962573], *Chrysemys_picta* [KF874616]), and five birds that represent three major divisions of Aves: Palaeognathae (*Struthio camelus* [NC_002785]), Galloanserae, (*Anas platyrhynchos* [EU755253], *Gallus gallus* [NC_007236], *Meleagris gallopavo* [NC_010195]), and Neoaves (*Melopsittacus undulatus* [NC_009134], *Taeniopygia guttata* [NC_007897]).

For the 33 extant taxa, mt genomes initially were aligned using MUSCLE^97^ in Geneious 8.1.9^98^. Minor adjustments were made to the alignment using Se-Al^99^ and genes were delimited based on published annotations. Several pairs of genes overlap each other in the mt genomes of Crocodylia. Therefore, each overlapping region was assigned to only one of the genes for the purposes of phylogenetic analyses. To maintain reading frame in all protein-coding genes, seven autapomorphic indels (each 1 bp) were deleted from the multi-species alignment (three in *Alligator sinensis*, two in *Crocodylus palustris*, two in *Pelodiscus sinensis*). A 1 bp insertion in the ND3 gene shared in turtles and birds corresponds to a site that is not translated and was also excluded from the final alignment. Two rDNA genes and 13 protein-coding genes were included in the final alignment. Each reconstructed *Voay* mt genome build (*Voay* 3101 *Osteolaemus* ref.; *Voay* AMNH FR-3103 *Osteolaemus* ref.; *Voay* AMNH FR-3101 *C. porosus* ref.; *Voay* AMNH FR-3103 *C. porosus* ref.) was incorporated into the multispecies mtDNA alignment by inserting gaps where there were alignment gaps in the particular reference genome used as template for mapping *Voay* sequencing reads. The final mtDNA alignment is available in Supplementary Data 1.

Parsimony analyses were performed using PAUP* 4.0a build 161^100^. Gaps were treated as missing data, all character state transformations were equally weighted, and the stability of results was assessed by weighting characters by relative fit. The concavity of the weighting function, k, was set at 4, 8, and 12 in successive runs with Goloboff weighting^101^. Searches were heuristic with 100 random taxon addition replicates and tree-bisection reconnection (TBR) branch swapping. Relative support was assessed by nonparametric bootstrapping^102^ with 100–1000 pseudoreplicates, and each search included 10 random taxon addition replicates.

ML analyses with bootstrapping (1000 replicates, gaps treated as missing data, randomized MP starting trees, and the fast hill-climbing algorithm with all free parameters estimated) were performed using RAxML-HPC v.8^103^ on XSEDE utilizing the Cipres portal^104,105^. When multiple data partitions were specified (individual genes, rDNAs, stems versus loops of rDNAs, protein-coding regions, three codon positions), different GTR + Γ models were permitted for each subset of characters^104^. RNAalifold^106^ with default settings was used to predict consensus rRNA secondary structures for aligned mt rDNA sequences.

Multiple searches were performed using both parsimony and ML optimality criteria to investigate the phylogenetic placements of the four mt genome builds for *Voay robustus*. In our primary mt genome tree based on ML analysis (Fig. 3), the four *Voay* mt genome builds were included and 15 character partitions (one for each mt gene) were analyzed. Variations in taxon sampling, character sampling, character weighting, and data partitioning were explored to assess the robustness of phylogenetic results for the placement of *Voay* relative to the major extant lineages of Crocodylia (Supplementary Table 1 and Data 3; Supplementary File 2).

We performed six tip-dating analyses using BEAST v1.8.3^107^ with modified versions of xml files provided by Lee and Yates^37^ (Supplementary Table 1 and Data 3; Supplementary Data 2). Lee and Yates assembled two combined datasets that both utilized published DNA sequences for crocodylians^37^. Their primary combined dataset included 278 morphological characters for 25 extant and 92 extinct taxa as well as stratigraphic data. They also assembled a second combined dataset that included 189 morphological characters for 15 extant and 85 extinct taxa from Brochu^46^ and stratigraphic data. The larger dataset in their study includes several new characters as well as modifications to characters and codings used in previous analyses; our close examination of this dataset reveals incorporated errors^36,108^, and results should thus be treated with caution.

In our six tip-dating analyses, we replaced the molecular data in these matrices with our mt protein-coding gene alignments that included the *Voay* AMNH FR-3101 *C. porosus* ref. build or the *Voay* AMNH FR-3101 *Osteolaemus* ref. build. The mt protein-coding data were partitioned by codon (1^st^, 2^nd^, 3^rd^ or 1^st^, 2^nd^ with 3^rd^ codons excluded), and each data partition was modeled under the GTR + G model of sequence evolution. Clock models for the different codon positions were unlinked. All model parameters for morphological data were set as in Lee and Yates^37^, and we utilized their xml files (“BEAST1.8.2_croc_117_9562_2ucln_NoMolecCal_NoAsc_AsPublished.xml” and “BEAST1.8.2_Brochu2013matrixPlusMolecules.xml”). For each tip-dating analysis, two to eight independent BEAST runs were implemented for 50 million generations each with sampling every 50,000 generations. In all BEAST runs, we used Tracer^109^ to determine burn-in and RWTY^110^ to test for parameter convergence. Based on these results, 20%-25% burn-in was chosen. All post-burn-in samples were combined with LogCombiner and TreeAnnotator for subsequent analyses. Tip-dating has the advantage of directly incorporating extensive fossil information in relaxed clock models, but does not account for incomplete lineage sorting, a process that can bias divergences toward older dates^58^.

We performed biogeographical mapping using equally weighted parsimony and minimum area change (MAC) parsimony^111^. Four broad geographical areas were defined (Africa, Madagascar, Australia/Asia, and the New World (Fig. 4). For our primary analysis, the BEAST MCC tree with the most taxa and molecular data for *Voay* was used (“BEAST1.8.2_croc_117_9562_2ucln_NoMolecCal_NoAsc_AsPublished.xml” with the *Voay* AMNH FR-3101 *C. porosus* reference build). Distantly related taxa were pruned from the overall topology (Fig. 4). Equally-weighted parsimony treated the four areas as unordered character states. MAC parsimony used a step-matrix with equal costs to gains and losses of geographic areas^111^. We utilized a step matrix that allows a maximum geographic range of two areas, the extreme observed for the taxa sampled in our tree. Biogeographic reconstructions for the remaining five tip-dated MCC timetrees (Supplementary Data 2) were used to assess the robustness of results.

Morphological synapomorphies for the placement of *Voay* were based on parsimony optimizations of phenotypic characters using PAUP*. Unequivocally optimized character state changes were mapped to alternative trees for Crocodylia to assess changes in character support that came with the addition of mtDNA data for *Voay* to the combined dataset published by Lee and Yates^37^. We mapped synapomorphic character state changes on our tip-dated MCC tree for the *Voay* + *Crocodylus* clade (Fig. 4) and recorded homoplastic evolution of the same characters on Lee and Yates’ tip-dated timetree. We also noted synapomorphies for the *Voay* + *Osteolaemus* clade in their tree and recorded homoplasy in these characters on our tree. Finally, the presence of squamosal “horns”, a distinctive cranial feature of *Voay* (Fig. 1), was optimized to infer the evolutionary history of this trait.

### Reporting summary

Further information on research design is available in the Nature Research Reporting Summary linked to this article.

## Supplementary information

Supplementary Information

Description of Additional Supplementary Files

Supplementary Data 1

Supplementary Data 2

Supplementary Data 3

Reporting Summary

## Data Availability

The paleontological collections at the American Museum of Natural History (AMNH) house the two specimens of *Voay* (= *Crocodylus*) *robustus* used in the study (AMNH FR-3101 and AMNH FR-3103). The short read data for each *Voay* specimen used in the analyses were deposited at the NCBI short read archive (SRA) under Bioproject PRJNA681754.

## References

[1] Willerslev E, Cooper A (2005). Ancient DNA. Proc. Biol. Sci..

[2] Vilstrup, J. T. et al. Mitochondrial phylogenomics of modern and ancient equids. *PLoS ONE* **8**, e55950, (2013). 10.1371/journal.pone.0055950. 10.1371/journal.pone.0055950 PMC3577844 23437078

[3] Enk JM (2014). Ancient whole genome enrichment using baits built from modern DNA. Mol. Biol. Evol..

[4] Grealy, A. et al. Eggshell palaeogenomics: Palaeognath evolutionary history revealed through ancient nuclear and mitochondrial DNA from Madagascan elephant bird (*Aepyornis* sp.) eggshell. *Mol. Phylogenet. Evol*. (2017). 10.1016/j.ympev.2017.01.005 10.1016/j.ympev.2017.01.005 28089793

[5] Mitchell, K. J. et al. Ancient DNA reveals elephant birds and kiwi are sister taxa and clarifies ratite bird evolution. *Science*. (2009). 10.3356/JRR-08-18.1 10.1126/science.1251981 24855267

[6] Yonezawa, T. et al. Phylogenomics and morphology of extinct paleognaths reveal the origin and evolution of the ratites. *Curr. Biol*. (2017). 10.1016/j.cub.2016.10.029 10.1016/j.cub.2016.10.029 27989673

[7] Kehlmaier, C. et al. Tropical ancient DNA reveals relationships of the extinct Bahamian giant tortoise *Chelonoidis alburyorum*. *Proc. R. Soc. B Biol. Sci*. (2017). 10.1098/rspb.2016.2235 10.1098/rspb.2016.2235 PMC5247498 28077774

[8] Grealy, A., McDowell, M., Retallick, C., Bunce, M. & Peacock, D. Novel mitochondrial haplotype of spotted-tailed quoll (*Dasyurus maculatus*) present on Kangaroo Island (South Australia) prior to extirpation. *Holocene* (2019). 10.1177/0959683619875805

[9] Green, R. E. et al. A draft sequence of the Neandertal genome. *Science*. (2010). 10.1126/science.1188021 10.1126/science.1188021 PMC5100745 20448178

[10] Vernot, B. et al. Excavating Neandertal and Denisovan DNA from the genomes of Melanesian individuals. *Science*. (2016). 10.1126/science.aad9416 10.1126/science.aad9416 PMC6743480 26989198

[11] Pilot, M. et al. Global Phylogeographic and admixture patterns in grey wolves and genetic legacy of an ancient Siberian lineage. *Sci. Rep*. (2019). 10.1038/s41598-019-53492-9 10.1038/s41598-019-53492-9 PMC6874602 31757998

[12] Palkopoulou, E. et al. A comprehensive genomic history of extinct and living elephants. *Proc. Natl. Acad. Sci. USA* (2018). 10.1073/pnas.1720554115 10.1073/pnas.1720554115 PMC5856550 29483247

[13] Kistler, L. et al. Comparative and population mitogenomic analyses of Madagascar’s extinct, giant “subfossil” lemurs. *J. Hum. Evol*. (2015). 10.1016/j.jhevol.2014.06.016 10.1016/j.jhevol.2014.06.016 25523037

[14] Kistler, L., Ware, R., Smith, O., Collins, M. & Allaby, R. G. A new model for ancient DNA decay based on paleogenomic meta-analysis. *Nucleic Acids Res*. (2017). 10.1093/nar/gkx361 10.1093/nar/gkx361 PMC5499742 28486705

[15] Crowley, B. E. A refined chronology of prehistoric Madagascar and the demise of the megafauna. *Quat. Sci. Rev*. (2010). 10.1016/j.quascirev.2010.06.030

[16] Goodman, S. M. & Jungers, W. L. A Brief History of Climatic Change on Madagascar since the Late Pleistocene. In *Extinct Madagascar: Picturing the island’s past* (2014).

[17] Crowley, B. E. et al. Island-wide aridity did not trigger recent megafaunal extinctions in Madagascar. *Ecography*. (2017). 10.1111/ecog.02376

[18] Godfrey, L. R. et al. A new interpretation of Madagascar’s megafaunal decline: The “Subsistence Shift Hypothesis”. *J. Hum. Evol*. (2019). 10.1016/j.jhevol.2019.03.002 10.1016/j.jhevol.2019.03.002 31010539

[19] Hansford, J. et al. Early Holocene human presence in Madagascar evidenced by exploitation of avian megafauna. *Sci. Adv*. (2018). 10.1126/sciadv.aat6925 10.1126/sciadv.aat6925 PMC6135541 30214938

[20] Wang L (2019). The African Humid Period, rapid climate change events, the timing of human colonization, and megafaunal extinctions in Madagascar during the Holocene: Evidence from a 2m Anjohibe Cave stalagmite. Quat. Sci. Rev..

[21] Austin, J. J., Arnold, E. N. & Bour, R. Was there a second adaptive radiation of giant tortoises in the Indian Ocean? Using mitochondrial DNA to investigate speciation and biogeography of *Aldabrachelys* (Reptilia, Testudinidae). *Mol. Ecol*. (2003). 10.1046/j.1365-294X.2003.01842.x 10.1046/j.1365-294x.2003.01842.x 12755871

[22] Mitchell, K. J., Wood, J. R., Scofield, R. P., Llamas, B. & Cooper, A. Ancient mitochondrial genome reveals unsuspected taxonomic affinity of the extinct Chatham duck (*Pachyanas chathamica*) and resolves divergence times for New Zealand and sub-Antarctic brown teals. *Mol. Phylogenet. Evol*. (2014). 10.1016/j.ympev.2013.08.017 10.1016/j.ympev.2013.08.017 23994164

[23] van der Geer, A. A. E., Lyras, G. A., Mitteroecker, P. & MacPhee, R. D. E. From Jumbo to Dumbo: Cranial shape changes in elephants and hippos during phyletic dwarfing. *Evol. Biol*. (2018). 10.1007/s11692-018-9451-1

[24] Vaillant, L., Grandidier, A. Histoire naturelle des reptiles: premier partie: Crocodiles et tortues. *Impr. Natl*. **17**, (1910).

[25] Vaillant L (1883). Remarques sur le *Crocodilus robustus*, Vaillant et Grandidier, de Madagascar. Compte Rendu Acad. Sci..

[26] King, F. W. & Burke, R. L. Crocodilians, tuatara, and turtle species of the world. *Assoc. Syst. Coll*. (1989).

[27] Grandidier Alfred, Vaillant L (1872). Sur le crocodile fossile d’Amboulistare (Madagascar). Compte Rendu Acad. Sci..

[28] Grandidier A (1872). Description des quleques reptiles noveaux decouverts a Madagascar en 1870. Ann. Sci. Natur. Paris.

[29] Barbour, T. Vertebrata from Madagascar; Amphibia, Reptilia. *Bull. Museum Comp. Zool*. 479–489 (1918).

[30] Boettger, O. *Die Reptilien und Amphibien von Madagascar*. (2011). 10.5962/bhl.title.9082

[31] Mook, C. C. Individual and age variations in the skulls of recent Crocodilia. *Bull. Am. Museum Nat. Hist*. (1921).

[32] Mook, C. Description of a skull of the extinct Madagascar crocodile, *Crocodylus robustus* Vaillant and Grandidier. *Am. Museum Novit*. (1921).

[33] Brochu CA (2007). Morphology, relationships, and biogeographical significance of an extinct horned crocodile (Crocodylia, Crocodylidae) from the Quaternary of Madagascar. Zool. J. Linn. Soc..

[34] Brochu, C. A., Njau, J., Blumenschine, R. J. & Densmore, L. D. A new horned crocodile from the Plio-Pleistocene hominid sites at Olduvai Gorge, Tanzania. *PLoS ONE* (2010). 10.1371/journal.pone.0009333 10.1371/journal.pone.0009333 PMC2827537 20195356

[35] Brochu, C. A. & Storrs, G. W. A giant crocodile from the Plio-Pleistocene of Kenya, the phylogenetic relationships of Neogene African crocodylines, and the antiquity of *Crocodylus* in Africa. *J. Vertebr. Paleontol*. (2012). 10.1080/02724634.2012.652324

[36] Conrad, J. L. et al. New specimens of *Crocodylus pigotti* (Crocodylidae) from Rusinga Island, Kenya, and generic reallocation of the species. *J. Vertebr. Paleontol*. (2013). 10.1080/02724634.2013.743404

[37] Lee, M. S. Y. & Yates, A. M. Tip-dating and homoplasy: Reconciling the shallow molecular divergences of modern gharials with their long fossil record. *Proc. R. Soc. B Biol. Sci.* (2018). 10.1098/rspb.2018.1071 10.1098/rspb.2018.1071 PMC6030529 30051855

[38] Salas-Gismondi, R., Moreno-Bernal, J. W., Scheyer, T. M., Sánchez-Villagra, M. R. & Jaramillo, C. New Miocene Caribbean gavialoids and patterns of longirostry in crocodylians. *J. Syst. Palaeontol*. **17**, (2019).

[39] Iijima, M. & Kobayashi, Y. Mosaic nature in the skeleton of East Asian crocodylians fills the morphological gap between “Tomistominae” and Gavialinae. *Cladistics* (2019). 10.1111/cla.12372 10.1111/cla.12372 34618925

[40] Gatesy, J., Amato, G., Norell, M., DeSalle, R. & Hayashi, C. Combined support for wholesale taxic atavism in gavialine crocodylians. *Syst. Biol*. (2003). 10.1080/10635150390197037 10.1080/10635150390197037 12775528

[41] Gatesy, J., Baker, R. H. & Hayashi, C. Inconsistencies in arguments for the supertree approach: Supermatrices versus supertrees of Crocodylia. *Syst. Biol.* (2004). 10.1080/10635150490423971 10.1080/10635150490423971 15205058

[42] Muldoon, K. M. et al. Early Holocene fauna from a new subfossil site: a first assessment from Christmas River, south central Madagascar. *Madagascar Conserv. Dev*. (2012). 10.4314/mcd.v7i1.5

[43] Gatesy, J. & Amato, G. The rapid accumulation of consistent molecular support for intergeneric crocodylian relationships. *Mol. Phylogenet. Evol*. (2008). 10.1016/j.ympev.2008.02.009 10.1016/j.ympev.2008.02.009 18372192

[44] Brochu, C. A. Morphology, fossils, divergence timing, and the phylogenetic relationships of *Gavialis*. *Syst. Biol*. (1997). 10.2307/2413693 10.1093/sysbio/46.3.479 11975331

[45] Mook, C. Skull characters of recent Crocodilia: With notes on the affinities of the recent genera. *Am. Museum Novit*. (1921).

[46] Brochu, C. A. Phylogenetic relationships of Palaeogene ziphodont eusuchians and the status of *Pristichampsus* Gervais, 1853. *Earth Environ. Sci. Trans. R. Soc. Edinburgh* (2013). 10.1017/S1755691013000200

[47] Densmore, L. D. Biochemical and immunological systematics of the Order Crocodilia. In *Evolutionary Biology* (1983). 10.1007/978-1-4615-6971-8_8

[48] Gatesy, J. & Amato, G. D. Sequence similarity of 12S ribosomal segment of mitochondrial DNAs of gharial and false gharial. *Copeia* (1992). 10.2307/1446560

[49] Iijima M (2017). Assessment of trophic ecomorphology in non-alligatoroid crocodylians and its adaptive and taxonomic implications. J. Anat..

[50] Ballell, A., Moon, B. C., Porro, L. B., Benton, M. J. & Rayfield, E. J. Convergence and functional evolution of longirostry in crocodylomorphs. *Palaeontology* (2019). 10.1111/pala.12432

[51] Drumheller, S. K. & Wilberg, E. W. A synthetic approach for assessing the interplay of form and function in the crocodyliform snout. *Zool. J. Linn. Soc*. (2019). 10.1093/zoolinnean/zlz081

[52] Felice, R. N. et al. Evolutionary integration and modularity in the archosaur cranium. In *Integrative and Comparative Biology* (2019). 10.1093/icb/icz052 10.1093/icb/icz052 31120528

[53] Godoy, P. L. Crocodylomorph cranial shape evolution and its relationship with body size and ecology. *J. Evol. Biol*. (2019). 10.1111/jeb.13540 10.1111/jeb.13540 31566848

[54] Groh, S. S., Upchurch, P., Barrett, P. M. & Day, J. J. The phylogenetic relationships of neosuchian crocodiles and their implications for the convergent evolution of the longirostrine condition. *Zool. J. Linn. Soc*. (2019). 10.1093/zoolinnean/zlz117

[55] Morris, Z. S., Vliet, K. A., Abzhanov, A. & Pierce, S. E. Heterochronic shifts and conserved embryonic shape underlie crocodylian craniofacial disparity and convergence. *Proc. R. Soc. B Biol. Sci*. (2019). 10.1098/rspb.2018.2389 10.1098/rspb.2018.2389 PMC6408887 30963831

[56] Sookias, R. B. Exploring the effects of character construction and choice, outgroups and analytical method on phylogenetic inference from discrete characters in extant crocodilians. *Zool. J. Linn. Soc*. (2019). 10.1093/zoolinnean/zlz015

[57] Wilberg, E. W. Phylogenetic and morphometric assessment of the evolution of the longirostrine crocodylomorphs. *Univ. Iowa* (2012).

[58] Oaks JR (2011). time-calibrated species tree of Crocodylia reveals a recent radiation of the true crocodiles. Evolution.

[59] Meredith RW, Hekkala ER, Amato G, Gatesy J (2011). A phylogenetic hypothesis for *Crocodylus* (Crocodylia) based on mitochondrial DNA: evidence for a trans-Atlantic voyage from Africa to the New World. Mol. Phylogenet. Evol..

[60] Hekkala E (2011). An ancient icon reveals new mysteries: Mummy DNA resurrects a cryptic species within the Nile crocodile. Mol. Ecol..

[61] Nicolaï, M. P. J. & Matzke, N. J. Trait-based range expansion aided in the global radiation of Crocodylidae. *Glob. Ecol. Biogeogr.* **28**, 1244–1258 (2019).

[62] Brochu, C. A. Phylogenetic relationships and divergence timing of *Crocodylus* based on morphology and the fossil record. *Copeia* (2000). 10.1643/0045-8511(2000)000[0657:pradto]2.0.co;2

[63] Jemvall, J., Wright, P. C., Ravoavy, F. L., Simons, E. L. Report on the findings of subfossils at Amposa and Ampanihy in Southwestern Madagascar. *Lemurs News* **8**, (2003).

[64] deMenocal PB (2004). African climate change and faunal evolution during the Pliocene-Pleistocene. Earth Planet. Sci. Lett..

[65] Samonds, K. E. et al. Spatial and temporal arrival patterns of Madagascar’s vertebrate fauna explained by distance, ocean currents, and ancestor type. *Proc. Natl. Acad. Sci. USA* (2012). 10.1073/pnas.1113993109 10.1073/pnas.1113993109 PMC3325680 22431643

[66] Samonds, K. E. et al. Imperfect isolation: factors and filters shaping Madagascar’s extant vertebrate fauna. *PLoS ONE* (2013). 10.1371/journal.pone.0062086 10.1371/journal.pone.0062086 PMC3633922 23626770

[67] Taplin LE, Grigg GC (1989). Historical zoogeography of the eusuchian crocodilians: a physiological perspective. Integr. Comp. Biol..

[68] Mazzotti, F. J. & Dunson, W. A. Adaptations of *Crocodylus acutus* and *Alligator* for life in saline water. *Comp. Biochem. Physiol. Part A Physiol*. (1984). 10.1016/0300-9629(84)90462-6

[69] Pidcock S, Taplin LE, Grigg GC (1997). Differences in renal-cloacal function between *Crocodylus porosus* and *Alligator mississippiensis* have implications for crocodilian evolution. J. Comp. Physiol. B Biochem. Syst. Environ. Physiol..

[70] Larsen PF, Nielsen EE, Williams TD, Loeschcke V (2008). Intraspecific variation in expression of candidate genes for osmoregulation, heme biosynthesis and stress resistance suggests local adaptation in European flounder (*Platichthys flesus*). Heredity.

[71] Leslie, A. J. & Spotila, J. R. Osmoregulation of the Nile crocodile, *Crocodylus niloticus*, in Lake St. Lucia, Kwazulu/Natal, South Africa. *Comp. Biochem. Physiol. A Mol. Integr. Physiol*. (2000). 10.1016/S1095-6433(00)00215-4 10.1016/s1095-6433(00)00215-4 10964030

[72] MacPhee, R. D. E., Burney, D. A. & Wells, N. A. Early Holocene chronology and environment of Ampasambazimba, a Malagasy subfossil lemur site. *Int. J. Primatol*. 10.1007/BF02735571 (1985).

[73] Burney, D. et al. Environmental change, extinction and human activity: Evidence from caves in NW Madagascar. *J. Biogeogr*. (2003). 10.1046/j.1365-2699.1997.00146.x

[74] Burney, D. A. et al. A chronology for late prehistoric Madagascar. *J. Hum. Evol*. (2004). 10.1016/j.jhevol.2004.05.005 10.1016/j.jhevol.2004.05.005 15288523

[75] Bickelmann, C. & Klein, N. The late Pleistocene horned crocodile *Voay robustus* (Grandidier & Vaillant, 1872) from Madagascar in the Museum für Naturkunde Berlin. *Foss. Rec*. (2009). 10.1002/mmng.200800007

[76] Chesson P (2000). Mechanisms of maintenance of species diversity. Annu. Rev. Ecol. Syst..

[77] Rubidge EM, Monahan WB, Parra JL, Cameron SE, Brashares JS (2011). The role of climate, habitat, and species co-occurrence as drivers of change in small mammal distributions over the past century. Glob. Chang. Biol..

[78] Dowell, S. A. & Hekkala, E. R. Divergent lineages and conserved niches: using ecological niche modeling to examine the evolutionary patterns of the Nile monitor (*Varanus niloticus*). *Evol. Ecol*. **30**, (2016).

[79] Shirley, M. H. & Austin, J. D. Did Late Pleistocene climate change result in parallel genetic structure and demographic bottlenecks in sympatric Central African crocodiles, *Mecistops* and *Osteolaemus*? *Mol. Ecol*. (2017). 10.1111/mec.14378 10.1111/mec.14378 29024142

[80] Mathews, J. C. & Samonds, K. E. A juvenile subfossil crocodylian from Anjohibe Cave, Northwestern Madagascar. *PeerJ* (2016). 10.7717/peerj.2296 10.7717/peerj.2296 PMC5028775 27672490

[81] Milián-García, Y., Ramos-Targarona, R., Pérez-Fleitas, E., Espinosa-López, G., & Russello, M. A. Genetic evidence of hybridization between the critically endangered Cuban crocodile and the American crocodile: implications for population history and in situ/ex situ conservation. *Heredity* (2015). 10.1038/hdy.2014.96 10.1038/hdy.2014.96 PMC4815585 25335559

[82] Rhymer, J. & Simberloff, D. Extinction by hybridization and introgression. *Annu. Rev. Ecol. Syst*. (1996). 10.1146/annurev.ecolsys.27.1.83

[83] Milián-García Y (2018). Genetic evidence supports a distinct lineage of American crocodile (*Crocodylus acutus*) in the Greater Antilles. PeerJ.

[84] Milián-García Y (2020). Phylogenomics reveals novel relationships among Neotropical crocodiles (*Crocodylus* spp.). Mol. Phylogenet. Evol..

[85] White, E. I. Fossil Hunting in Madagascar. *Natural History Magazine London* 209–235 (1930).

[86] Goodman, S. M., Jungers, W. L. & Simeonovski, V. *Extinct Madagascar* (2019). 10.7208/chicago/9780226156941.001.0001

[87] Gamba, C. et al. Comparing the performance of three ancient DNA extraction methods for high-throughput sequencing. *Mol. Ecol. Resour*. (2016). 10.1111/1755-0998.12470 10.1111/1755-0998.12470 26401836

[88] Carpenter ML (2013). Pulling out the 1%: Whole-Genome capture for the targeted enrichment of ancient DNA sequencing libraries. Am. J. Hum. Genet..

[89] Peltzer A (2016). EAGER: efficient ancient genome reconstruction. Genome Biol..

[90] Knapp M, Hofreiter M (2010). Next generation sequencing of ancient DNA: requirements, strategies and perspectives. Genes.

[91] Günther, T. & Nettelblad, C. The presence and impact of reference bias on population genomic studies of prehistoric human populations. *PLoS Genet*. (2019). 10.1371/journal.pgen.1008302 10.1371/journal.pgen.1008302 PMC6685638 31348818

[92] Eaton MJ, Martin A, Thorbjarnarson J, Amato G (2009). Species-level diversification of African dwarf crocodiles (Genus *Osteolaemus*): A geographic and phylogenetic perspective. Mol. Phylogenet. Evol..

[93] Shirley MH, Vliet KA, Carr AN, Austin JD (2014). Rigorous approaches to species delimitation have significant implications for African crocodilian systematics and conservation. Proc. Biol. Sci..

[94] Shirley, M. H., Carr, A. N., Nestler, J. H., Vliet, K. A. & Brochu, C. A. Systematic revision of the living African slender-snouted crocodiles (*Mecistops* Gray, 1844). *Zootaxa* (2018). 10.11646/zootaxa.4504.2.1 10.11646/zootaxa.4504.2.1 30486023

[95] Gauthier, J., Kluge, A. G. & Rowe, T. Amniote phylogeny and the importance of fossils. *Cladistics* (1988). 10.1111/j.1096-0031.1988.tb00514.x 10.1111/j.1096-0031.1988.tb00514.x 34949076

[96] Chiari, Y., Cahais, V., Galtier, N. & Delsuc, F. Phylogenomic analyses support the position of turtles as the sister group of birds and crocodiles (Archosauria). *BMC Biol*. (2012) 10.1186/1741-7007-10-65 10.1186/1741-7007-10-65 PMC3473239 22839781

[97] Edgar, R. C. MUSCLE: Multiple sequence alignment with high accuracy and high throughput. *Nucleic Acids Res*. (2004). 10.1093/nar/gkh340 10.1093/nar/gkh340 PMC390337 15034147

[98] Meintjes, P. et al. Geneious Basic: An integrated and extendable desktop software platform for the organization and analysis of sequence data. *Bioinformatics* (2012). 10.1093/bioinformatics/bts199 PMC3371832 22543367

[99] Rambaut, A. Se-Al: Sequence alignment editor, 2.0a11. (1996). http://evolve.zoo.ox.ac.uk

[100] Swofford, D. L. Phylogenetic Analysis Using Parsimony * (and other methods). Version 4. *Options* (2002). 10.1159/000170955

[101] Goloboff, P. A. Estimating character weights during tree search. *Cladistics* (1993). 10.1111/j.1096-0031.1993.tb00209.x 10.1111/j.1096-0031.1993.tb00209.x 34929936

[102] Felsenstein, J. Confidence limits on phylogenies: An approach using the bootstrap. *E**volution* (1985). 10.2307/2408678 10.1111/j.1558-5646.1985.tb00420.x 28561359

[103] Stamatakis A (2014). RAxML version 8: A tool for phylogenetic analysis and post-analysis of large phylogenies. Bioinformatics.

[104] Miller, M. A., Pfeiffer, W. & Schwartz, T. Creating the CIPRES Science Gateway for inference of large phylogenetic trees. In *2010 Gateway Computing Environments Workshop, GCE 2010* (2010). 10.1109/GCE.2010.5676129

[105] Abadi, S., Azouri, D., Pupko, T., Mayrose, I. Model selection may not be a mandatory step for phylogeny reconstruction. *Nat. Commun.* (2019). 10.1038/s41467-019-08822-w 10.1038/s41467-019-08822-w PMC6389923 30804347

[106] Bernhart, S. H., Hofacker, I. L., Will, S., Gruber, A. R., & Stadler, P. F. RNAalifold: Improved consensus structure prediction for RNA alignments. *BMC Bioinformatics* (2008). 10.1186/1471-2105-9-474 10.1186/1471-2105-9-474 PMC2621365 19014431

[107] Li, W. L. S. & Drummond, A. J. Model averaging and Bayes factor calculation of relaxed molecular clocks in Bayesian phylogenetics. *Mol. Biol. Evol*. (2012). 10.1093/molbev/msr232 10.1093/molbev/msr232 PMC3258040 21940644

[108] Cossette AP (2020). A new crocodylid from the middle Miocene of Kenya and the timing of crocodylian faunal change in the late Cenozoic of Africa. J. Paleontol..

[109] Rambaut, A. & Drummond, A. J. Tracer v1.4. *Encycl. Atmos. Sci*. (2007). 10.1017/CBO9781107415324.004

[110] Warren, D. L., Geneva, A. J., Lanfear, R. & Rosenberg, M. RWTY (R We There Yet): An R package for examining convergence of Bayesian phylogenetic analyses. *Mol. Biol. Evol*. (2017). 10.1093/molbev/msw279 10.1093/molbev/msw279 28087773

[111] Springer, M. S., Meredith, R. W., Janecka, J. E., & Murphy, W. J. The historical biogeography of Mammalia. *Philos. Trans. R. Soc. B Biol. Sci.* (2011). 10.1098/rstb.2011.0023 10.1098/rstb.2011.0023 PMC3138613 21807730

