# Author Correction: Paleogenomics illuminates the evolutionary history of the extinct Holocene “horned” crocodile of Madagascar, *Voay robustus*

**DOI:** 10.1038/s42003-021-02216-9

**Published:** 2021-05-25

**Authors:** E. Hekkala, J. Gatesy, A. Narechania, R. Meredith, M. Russello, M. L. Aardema, E. Jensen, S. Montanari, C. Brochu, M. Norell, G. Amato

**Affiliations:** 1 Department of Biological Sciences, Fordham University, Bronx, NY USA; 2 American Museum of Natural History, New York, NY USA; 3 Montclair State University, Montclair, NJ USA; 4 University of British Columbia, Department of Biology, Kelowna, BC Canada; 5 Newcastle University, School of Natural and Environmental Sciences Ecology Group, Newcastle, UK; 6 University of Iowa, Department of Geosciences, Iowa City, IA USA

**Keywords:** Palaeontology, Zoology

Correction to: *Communications Biology* 10.1038/s42003-021-02017-0, published online 27 April 2021.

In the original version of the Article, reference 61 was incorrectly given as: “Nikolai. Trait-based range expansion aided in the global radiation of Crocodylidae (2019).”

This has been corrected in the HTML and PDF versions of the Article to: “Nicolaï, M. P. J. and Matzke, N. J. Trait-based range expansion aided in the global radiation of Crocodylidae. *Glob. Ecol. Biogeogr*. **28**, 1244–1258 (2019).”

In addition, the reference to “Nikolai and Matzke” in the third paragraph of the Discussion has been corrected to “Nicolaï and Matzke” in the text in the HTML and PDF versions of the Article.

