# Supplementary material for: Paleogenomics illuminates the evolutionary history of the extinct Holocene “horned” crocodile of Madagascar, Voay robustus
Source: Commun Biol. 2021 Apr 27;4:505. doi: 10.1038/s42003-021-02017-0 (PMC8079395; doi:10.1038/s42003-021-02017-0)
Supplement: Supplementary file 1 — Supplementary Information [file 42003_2021_2017_MOESM1_ESM.pdf]

Supplementary Figures and Tables for Hekkala et al. 2021 Paleogenomics illuminates the evolutionary history of the extinct Holocene "horned" crocodile of Madagascar, *Voay robustus*

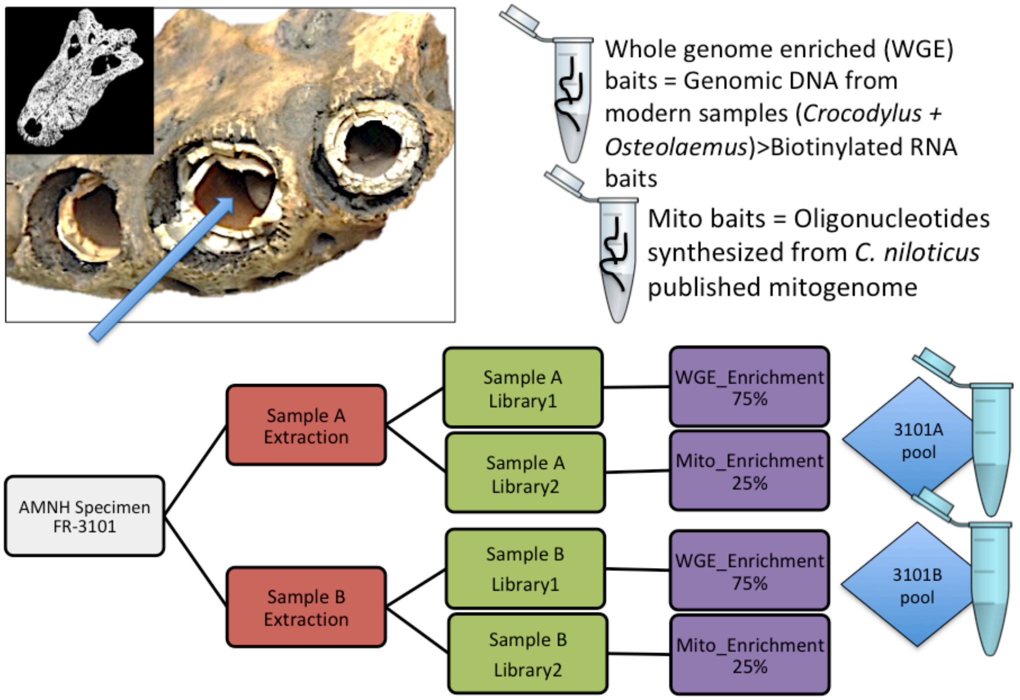

Supplementary Figure 1.
Schematic representation of sample processing from sampling to sequencing for subfossil skull of *Voay robustus* (AMNH FR-3101). Negative controls are not depicted.

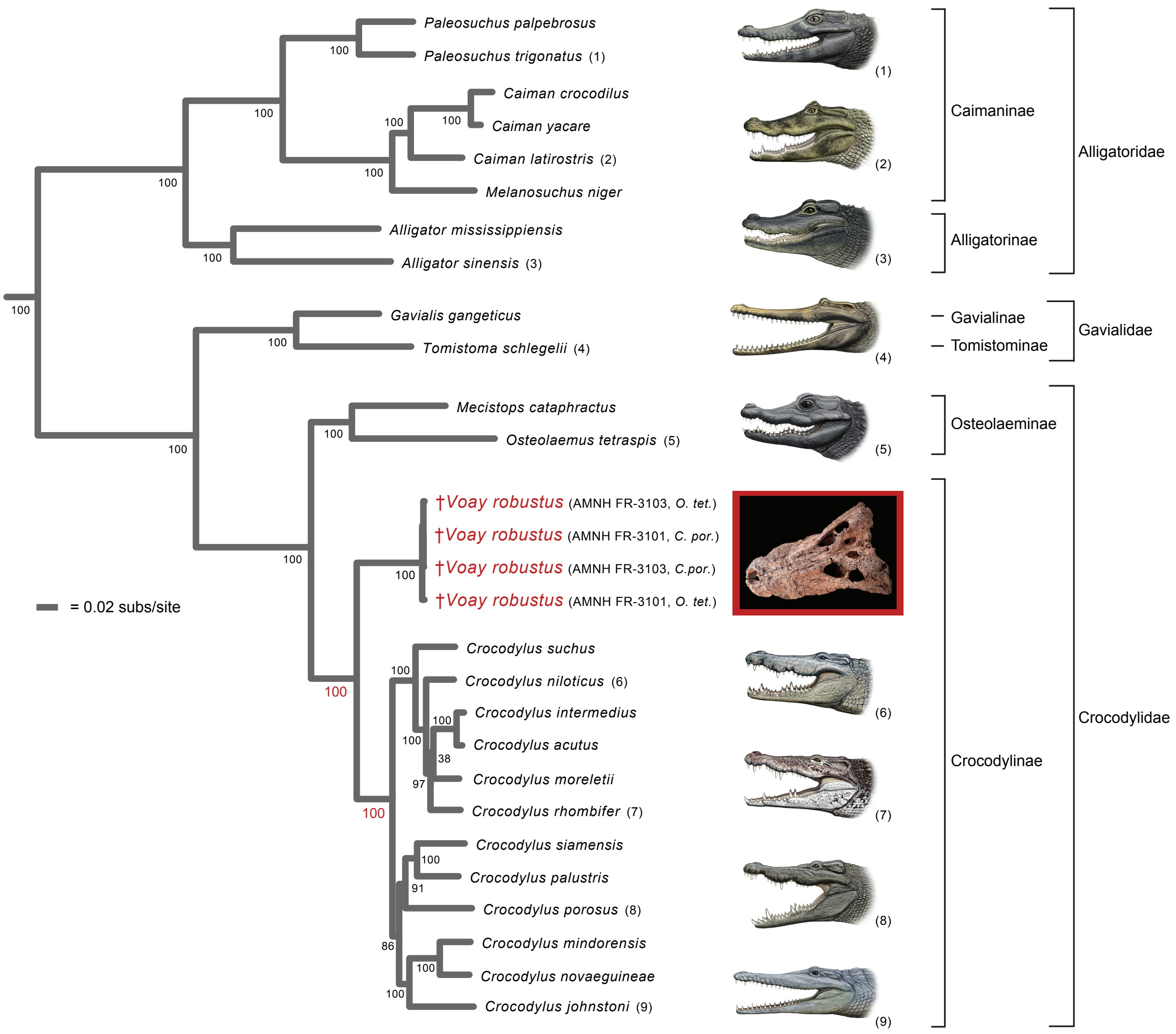

Supplementary Figure 2.
Phylogenetic relationships of *Voay robustus* (red) based on partial mitochondrial (mt) genomes with estimated branch lengths in expected substitutions per site. Analyses of mt data robustly and consistently support a sister group relationship between *Voay* and a monophyletic *Crocodylus* (true crocodiles). The ML phylogram in this figure is based on the full mtDNA data set (partitioned by gene) with all four builds of the *Voay* mt genome (*Voay* AMNH FR-3101 *C. porosus* reference build, *Voay* AMNH FR-3101 *Osteolaemus* reference build, *Voay* AMNH FR-3103 *C. porosus* reference build and *Voay* AMNH FR-3103 *Osteolaemus* reference build). Note the very short terminal branches for the four mt genome builds for the *Voay* specimens. The tree is rooted with bird, turtle, and lizard outgroups (not shown). Paintings of crocodylians are by C. Buell; photo of *Voay* skull (AMNH FR-3101) is by E. Hekkala.

Supplementary Table 1: RAxML maximum likelihood analyses, PAUP* parsimony analyses, and BEAST Bayesian tip-dating analyses used to test the stability of phylogenetic results. Trees with support scores can be found in Supplementary Data 2.

Data Set	*Voay* mt genome builds included in analysis	Partitioning Scheme

RAxML maximum likelihood analysis

rDNA, protein-coding		
	4 reference builds	No partitions
	3101 *Osteolaemus* reference build	No partitions
	3101 *C. porosus* reference build	No partitions
	3103 *Osteolaemus* reference build	No partitions
	3103 *C. porosus* reference build	No partitions
	4 reference builds	12S, 16S, ATP6, ATP8, COX1, COX2, COX3, ND1, ND2, ND3, ND4, ND4L, ND5, ND6, CYB
	3101 *Osteolaemus* reference build	12S, 16S, ATP6, ATP8, COX1, COX2, COX3, ND1, ND2, ND3, ND4, ND4L, ND5, ND6, CYB
	3101 *C. porosus* reference build	12S, 16S, ATP6, ATP8, COX1, COX2, COX3, ND1, ND2, ND3, ND4, ND4L, ND5, ND6, CYB
	3103 *Osteolaemus* reference build	12S, 16S, ATP6, ATP8, COX1, COX2, COX3, ND1, ND2, ND3, ND4, ND4L, ND5, ND6, CYB
	3103 *C. porosus* reference build	12S, 16S, ATP6, ATP8, COX1, COX2, COX3, ND1, ND2, ND3, ND4, ND4L, ND5, ND6, CYB
	4 reference builds	12S 16S stems, 12S 16S loops, ATP6, ATP8, COX1, COX2, COX3, ND1, ND2, ND3, ND4, ND4L, ND5, ND6, CYB
	3101 *Osteolaemus* reference build	12S 16S stems, 12S 16S loops, ATP6, ATP8, COX1, COX2, COX3, ND1, ND2, ND3, ND4, ND4L, ND5, ND6, CYB
	3101 *C. porosus* reference build	12S 16S stems, 12S 16S loops, ATP6, ATP8, COX1, COX2, COX3, ND1, ND2, ND3, ND4, ND4L, ND5, ND6, CYB

	3103 *Osteolaemus* reference build	12S 16S stems, 12S 16S loops, ATP6, ATP8, COX1, COX2, COX3, ND1, ND2, ND3, ND4, ND4L, ND5, ND6, CYB
	3103 *C. porosus* reference build	12S 16S stems, 12S 16S loops, ATP6, ATP8, COX1, COX2, COX3, ND1, ND2, ND3, ND4, ND4L, ND5, ND6, CYB
	4 reference builds	12S, 16S, 1st, 2nd, 3rd
	3101 *Osteolaemus* reference build	12S, 16S, 1st, 2nd, 3rd
	3101 *C. porosus* reference build	12S, 16S, 1st, 2nd, 3rd
	3103 *Osteolaemus* reference build	12S, 16S, 1st, 2nd, 3rd
	3103 *C. porosus* reference build	12S, 16S, 1st, 2nd, 3rd
	4 reference builds	12S 16S stems, 12S 16S loops, 1st, 2nd, 3rd
	3101 *Osteolaemus* reference build	12S 16S stems, 12S 16S loops, 1st, 2nd, 3rd
	3101 *C. porosus* reference build	12S 16S stems, 12S 16S loops, 1st, 2nd, 3rd
	3103 *Osteolaemus* reference build	12S 16S stems, 12S 16S loops, 1st, 2nd, 3rd
	3103 *C. porosus* reference build	12S 16S stems, 12S 16S loops, 1st, 2nd, 3rd
rDNA, protein-coding (no 3rd codons)		
	4 reference builds	12S 16S stems, 12S 16S loops, 1st, 2nd
	3101 *Osteolaemus* reference build	12S 16S stems, 12S 16S loops, 1st, 2nd
	3101 *C. porosus* reference build	12S 16S stems, 12S 16S loops, 1st, 2nd
	3103 *Osteolaemus* reference build	12S 16S stems, 12S 16S loops, 1st, 2nd
	3103 *C. porosus* reference build	12S 16S stems, 12S 16S loops, 1st, 2nd
protein-coding		
	4 reference builds	No partitions
	3101 *Osteolaemus* reference build	No partitions
	3101 *C. porosus* reference build	No partitions
	3103 *Osteolaemus* reference build	No partitions

	3103 *C. porosus* reference build	No partitions
	4 reference builds	ATP6, ATP8, COX1, COX2, COX3, ND1, ND2, ND3, ND4, ND4L, ND5, ND6, CYB
	3101 *Osteolaemus* reference build	ATP6, ATP8, COX1, COX2, COX3, ND1, ND2, ND3, ND4, ND4L, ND5, ND6, CYB
	3101 *C. porosus* reference build	ATP6, ATP8, COX1, COX2, COX3, ND1, ND2, ND3, ND4, ND4L, ND5, ND6, CYB
	3103 *Osteolaemus* reference build	ATP6, ATP8, COX1, COX2, COX3, ND1, ND2, ND3, ND4, ND4L, ND5, ND6, CYB
	3103 *C. porosus* reference build	ATP6, ATP8, COX1, COX2, COX3, ND1, ND2, ND3, ND4, ND4L, ND5, ND6, CYB
protein-coding (no 3rd codons)		
	4 reference builds	1st, 2nd
	3101 *Osteolaemus* reference build	1st, 2nd
	3101 *C. porosus* reference build	1st, 2nd
	3103 *Osteolaemus* reference build	1st, 2nd
	3103 *C. porosus* reference build	1st, 2nd
rDNA	4 reference builds	No partitions
	3101 *Osteolaemus* reference build	No partitions
	3101 *C. porosus* reference build	No partitions
	3103 *Osteolaemus* reference build	No partitions
	3103 *C. porosus* reference build	No partitions
	4 reference builds	12S, 16S
	3101 *Osteolaemus* reference build	12S, 16S
	3101 *C. porosus* reference build	12S, 16S
	3103 *Osteolaemus* reference build	12S, 16S
	3103 *C. porosus* reference build	12S, 16S

	4 reference builds	12S 16S stems, 12S 16S loops
	3101 *Osteolaemus* reference build	12S 16S stems, 12S 16S loops
	3101 *C. porosus* reference build	12S 16S stems, 12S 16S loops
	3103 *Osteolaemus* reference build	12S 16S stems, 12S 16S loops
	3103 *C. porosus* reference build	12S 16S stems, 12S 16S loops

PAUP* parsimony analysis (equal weighting and Goloboff weighting with k=4, 8, or 12)

rDNA, protein-coding	4 reference builds	No partitions

BEAST tip-dating analyses (Lee and Yates morphology matrix)

protein-coding	3101 *C. porosus* reference build	1st, 2nd, 3rd
	3101 *Osteolaemus* reference build	1st, 2nd, 3rd

protein-coding (no 3rd codons)	3101 *C. porosus* reference build	1st, 2nd
	3101 *Osteolaemus* reference build	1st, 2nd

BEAST tip-dating analyses (Brochu morphology matrix)

protein-coding	3101 *C. porosus* reference build	1st, 2nd, 3rd

protein-coding (no 3rd codons)	3101 *C. porosus* reference build	1st, 2nd
