# Supplementary material for: Paleogenomics illuminates the evolutionary history of the extinct Holocene “horned” crocodile of Madagascar, Voay robustus
Source: Commun Biol. 2021 Apr 27;4:505. doi: 10.1038/s42003-021-02017-0 (PMC8079395; doi:10.1038/s42003-021-02017-0)
Supplement: Supplementary file 2 — Description of Additional Supplementary Files [file 42003_2021_2017_MOESM2_ESM.pdf]

Description of Additional Supplementary Files

File name: Supplementary Data 1

Description: Voay mtDNA alignment nexus file

File name: Supplementary Data 2

Description: Voay RAXML BEAST trees text file

File name: Supplementary Data 3

Description: RAxML maximum likelihood analyses, PAUP* parsimony analyses, and BEAST Bayesian tip-dating analyses used to test the stability of phylogenetic results. Trees with support scores can be found in Supplementary Data 2.
