# Supplementary material for: Paleogenomics illuminates the evolutionary history of the extinct Holocene “horned” crocodile of Madagascar, Voay robustus
Source: Commun Biol. 2021 Apr 27;4:505. doi: 10.1038/s42003-021-02017-0 (PMC8079395; doi:10.1038/s42003-021-02017-0)
Supplement: Supplementary file 6 — Reporting Summary [file 42003_2021_2017_MOESM6_ESM.pdf]

Corresponding author(s): *Double-blind peer review submissions: write DBPR and your manuscript number here instead of author names.*

Last updated by author(s): 02/28/2021

Reporting Summary

Nature Research wishes to improve the reproducibility of the work that we publish. This form provides structure for consistency and transparency in reporting. For further information on Nature Research policies, see our Editorial Policies and the Editorial Policy Checklist.

Statistics

For all statistical analyses, confirm that the following items are present in the figure legend, table legend, main text, or Methods section.

n/a	Confirmed	
☐	☒	The exact sample size (n) for each experimental group/condition, given as a discrete number and unit of measurement
☐	☒	A statement on whether measurements were taken from distinct samples or whether the same sample was measured repeatedly
☒	☐	The statistical test(s) used AND whether they are one- or two-sided<br>*Only common tests should be described solely by name; describe more complex techniques in the Methods section.*
☒	☐	A description of all covariates tested
☒	☐	A description of any assumptions or corrections, such as tests of normality and adjustment for multiple comparisons
☒	☐	A full description of the statistical parameters including central tendency (e.g. means) or other basic estimates (e.g. regression coefficient) AND variation (e.g. standard deviation) or associated estimates of uncertainty (e.g. confidence intervals)
☒	☐	For null hypothesis testing, the test statistic (e.g. F, t, r) with confidence intervals, effect sizes, degrees of freedom and P value noted<br>*Give P values as exact values whenever suitable.*
☐	☒	For Bayesian analysis, information on the choice of priors and Markov chain Monte Carlo settings
☒	☐	For hierarchical and complex designs, identification of the appropriate level for tests and full reporting of outcomes
☒	☐	Estimates of effect sizes (e.g. Cohen's d, Pearson's r), indicating how they were calculated

Our web collection on statistics for biologists contains articles on many of the points above.

Software and code

Policy information about availability of computer code

Data collection	References are provided in the text and the reference section for all accessioned data used in the bioinformatic and phylogenetic analyses used the study.
Data analysis	References are provided in the text and the reference section for all code used in the bioinformatic and phylogenetic analysis pipelines used the study.

For manuscripts utilizing custom algorithms or software that are central to the research but not yet described in published literature, software must be made available to editors and reviewers. We strongly encourage code deposition in a community repository (e.g. GitHub). See the Nature Research guidelines for submitting code & software for further information.

Data

Policy information about availability of data

All manuscripts must include a data availability statement. This statement should provide the following information, where applicable:

- Accession codes, unique identifiers, or web links for publicly available datasets
- A list of figures that have associated raw data
- A description of any restrictions on data availability

The paleontological collections at the American Museum of Natural History (AMNH) house the two specimens of Voay (= Crocodylus) robustus used in the study (AMNH FR-3101 and AMNH FR-3103). The short read data for each Voay specimen used in the analyses were deposited at the NCBI short read archive (SRA) under Bioproject PRJNA681754

Field-specific reporting

Please select the one below that is the best fit for your research. If you are not sure, read the appropriate sections before making your selection.

☐ Life sciences ☐ Behavioural & social sciences ☒ Ecological, evolutionary & environmental sciences

For a reference copy of the document with all sections, see nature.com/documents/nr-reporting-summary-flat.pdf

Ecological, evolutionary & environmental sciences study design

All studies must disclose on these points even when the disclosure is negative.

Study description	We recovered partial mitochondrial (mt) genomes for 1300-1400 year old specimens (n=2) of the extinct "horned" crocodile, Voay robustus, collected from Holocene deposits in southwestern Madagascar. Phylogenetic analyses of these partial mt genomes and tip-dated timetrees based on molecular, fossil, and stratigraphic data favor a sister group relationship between Voay and Crocodylus (true crocodiles).
Research sample	The paleontological collections at the American Museum of Natural History (AMNH) include a series of specimens of Voay (= Crocodylus) robustus from southwestern Madagascar at Ampoza (44° 42.3' E, 22° 18.9' S, 570 m elevation) that were collected during the joint Mission Franco-Anglo-American expedition from 1927-193085. Two specimens, AMNH FR-3101 and AMNH FR-3103, that represent nearly complete crania from the Ampoza excavations, were targeted as potential sources of ancient DNA.
Sampling strategy	The sampling plan was designed to minimize damage to the specimens and reduce contamination. Subsampling of the two tooth specimens (AMNH FR-3101 and AMNH FR-3103) was done at the AMNH, and duplicate samples were shipped to the University of British Columbia (UBC). Isolation of ancient DNA was replicated in dedicated clean room facilities at the AMNH and at UBC (Suppl. Online Fig. 1) according to published protocols. Two duplicate Illumina® libraries for each Voay specimen were prepared in ancient DNA processing facilities by Arbor Biosciences for use in downstream WGE and targeted sequence capture of mtDNA.
Data collection	Enrichment of Voay robustus genomic libraries was conducted at Arbor Biosciences according to their MYcroarray capture protocol version 3 (https:// arborbiosci.com/wp-content/uploads/2017/10/MYbaits-manual-v3.pdf). For each of the two Voay robustus specimens (AMNH FR-3101 and AMNH FR-3103), two independent samples plus negative controls were extracted (A and B), two replicate libraries were produced (1 and 2) and one pooled WGE and Mito enriched library were sequenced, resulting in 10 separately processed samples. For each specimen replicate set (either 3101 or 3103), the indexed whole genome enriched library and the targeted mtDNA enriched library were pooled with a ratio of 75 (WGE library)/25 (mtDNA capture library), and sequenced using one full lane on an Illumina HiSeq® 2500 (paired-end, 150bp reads) at the New York Genome Center (see Suppl. Online Fig. 1 for sample AMNH FR-3101example).
Timing and spatial scale	Initial sampling of specimens was conducted in October 2014. Subsequent re-sampling and replications were conducted in December 2017. Analyses were completed in December 2019 and re-analyses were completed in May-August 2020.
Data exclusions	One sample was sequenced but the resulting data were not included in the final paper because of the difficulty of authenticating the source of the specimen. The sample was a small rib fragment with no diagnostic characters to identify it as belonging to the focal species Voay robustus.
Reproducibility	two independent samples plus negative controls were extracted (A and B), two replicate libraries were produced (1 and 2) and one pooled WGE and Mito enriched library were sequenced, resulting in 10 separately processed samples.
Randomization	two independent samples plus negative controls were extracted (A and B), two replicate libraries were produced (1 and 2) and one pooled WGE and Mito enriched library were sequenced, resulting in 10 separately processed samples.
Blinding	Genomic DNAs were sent to Arbor Biosciences with labels that did not indicate sample type (ie. whether positive or negative control or which replicate). All replicated and control samples were processed using identical protocols for sample preparation and sequencing with no prior knowledge of which sample contained which type.

Did the study involve field work? ☐ Yes ☒ No

Reporting for specific materials, systems and methods

We require information from authors about some types of materials, experimental systems and methods used in many studies. Here, indicate whether each material, system or method listed is relevant to your study. If you are not sure if a list item applies to your research, read the appropriate section before selecting a response.

Materials & experimental systems

n/a	Involved in the study
☒	☐ Antibodies
☒	☐ Eukaryotic cell lines
☐	☒ Palaeontology and archaeology
☒	☐ Animals and other organisms
☒	☐ Human research participants
☒	☐ Clinical data
☒	☐ Dual use research of concern

Methods

n/a	Involved in the study
☒	☐ ChIP-seq
☒	☐ Flow cytometry
☒	☐ MRI-based neuroimaging

Palaeontology and Archaeology

Specimen provenance	The paleontological collections at the American Museum of Natural History (AMNH) include a series of specimens of Voay (= Crocodylus) robustus from southwestern Madagascar at Ampoza (44° 42.3' E, 22° 18.9' S, 570 m elevation) that were collected during the joint Mission Franco-Anglo-American expedition from 1927-1930.
Specimen deposition	Two specimens are accessioned at the American Museum of Natural History, New York, NY in the Division of Vertebrate Paleontology (AMNH FR-3101 and AMNH FR-3103) and represent nearly complete crania from excavations conducted at Ampoza, Madagascar during the joint Mission Franco-Anglo-American expedition from 1927-1930.
Dating methods	Samples from each specimen (AMNH FR-3101 and AMNH FR-3103) were sent to Beta Analytic Inc, Miami Florida for radiocarbon dating. Teeth were initially decalcified and gelatinized using EDTA and HCl. Once collagen preservation was confirmed, samples were radiocarbon dated and calibrated dates reported. Calibration was calculated using one of the databases associated with the 2013 INTCAL program. Conventional Radiocarbon Ages and Sigmas are rounded to the nearest 10 years per the conventions of the 1977 International Radiocarbon Conference. When counting statistics produce Sigmas lower than +/- 30 years, a conservative +/- 30 BP is cited for the result. All work was performed under strict chain of custody and quality control under ISO/IEC 17025:2005 Testing Accreditation PJLA #59423 accreditation protocols. Sample, modern and blanks were all analyzed in the same chemistry lines by qualified professional technicians using identical reagents and counting parameters within on Beta Analytic Inc's own particle accelerators.

☒ Tick this box to confirm that the raw and calibrated dates are available in the paper or in Supplementary Information.

Ethics oversight	Specimen collection, sampling and sequencing were conducted under research ethics guidelines at the American Museum of Natural History.

Note that full information on the approval of the study protocol must also be provided in the manuscript.
